# Rostral Anterior Cingulate Cortex–Ventrolateral Periaqueductal Gray Circuit Underlies Electroacupuncture to Alleviate Hyperalgesia but Not Anxiety-Like Behaviors in Mice With Spared Nerve Injury

**DOI:** 10.3389/fnins.2021.757628

**Published:** 2022-01-12

**Authors:** Xixiao Zhu, Yingling Xu, Zui Shen, Haiyan Zhang, Siqi Xiao, Yichen Zhu, Mengwei Wu, Yeqing Chen, Zemin Wu, Yunyun Xu, Xiaofen He, Boyu Liu, Jinggen Liu, Junying Du, Jing Sun, Jianqiao Fang, Xiaomei Shao

**Affiliations:** Key Laboratory of Acupuncture and Neurology of Zhejiang Province, Department of Neurobiology and Acupuncture Research, The Third Clinical Medical College, Zhejiang Chinese Medical University, Hangzhou, China

**Keywords:** chronic pain, neuropathic pain, anxiety, rostral anterior cingulated cortex, periaqueductal gray, electroacupuncture

## Abstract

Neuropathic pain is a common cause of chronic pain and is often accompanied by negative emotions, making it complex and difficult to treat. However, the neural circuit mechanisms underlying these symptoms remain unclear. Herein, we present a novel pathway associated with comorbid chronic pain and anxiety. Using chemogenetic methods, we found that activation of glutamatergic projections from the rostral anterior cingulate cortex (rACC*^Glu^*) to the ventrolateral periaqueductal gray (vlPAG) induced both hyperalgesia and anxiety-like behaviors in sham mice. Inhibition of the rACC*^Glu^*-vlPAG pathway reduced anxiety-like behaviors and hyperalgesia in the spared nerve injury (SNI) mice model; moreover, electroacupuncture (EA) effectively alleviated these symptoms. Investigation of the related mechanisms revealed that the chemogenetic activation of the rACC*^Glu^*-vlPAG circuit effectively blocked the analgesic effect of EA in the SNI mice model but did not affect the chronic pain-induced negative emotions. This study revealed a novel pathway, the rACC*^Glu^*-vlPAG pathway, that mediates neuropathic pain and pain-induced anxiety.

## Introduction

Chronic pain is a major public health problem worldwide, with an incidence of 20–25% (López-González et al., 2017). Neuropathic pain caused by a lesion or disease of the somatosensory nervous system is a common cause of chronic pain and is characterized by spontaneous pain, shooting pain, and evoked amplified pain responses after noxious or non-noxious stimuli (Jensen and Finnerup, 2014). Deleterious changes in the central nervous system leads to high rates of comorbid neuropathic pain with psychological conditions, such as anxiety, depression, and sleep disorders (Descalzi et al., 2017; Guimarães et al., 2019). Negative emotions have been reported to exacerbate chronic pain, resulting in refractory diseases (Rhudy and Meagher, 2000; Ericsson et al., 2002). However, the neural circuit mechanisms underlying these symptoms remain unclear. Inadequate treatment of neuropathic pain is a major problem; hence, a better understanding of the mechanisms underlying the interaction between pain and pain-related negative emotions will facilitate the development of more constructive therapies (Baron et al., 2010).

The mechanisms underlying comorbid neuropathic pain and pain-related negative emotions are complicated; these include changes in a myriad of neurotransmitters and related substances, along with long-term maintenance of central sensitization and changes in neuroplasticity (Yang et al., 2018). The brain regions involved in neuropathic pain and emotional disorders, which make it difficult to treat neuropathic pain and pain-related negative emotions, should be studied in more detail (Benedetti et al., 2006; Suarez-Roca et al., 2008). Owing to the central neural anatomical connection, neuropathic pain may share the same neural pathway for pain and pain-related negative emotions (Zhou et al., 2019; Jin et al., 2020).

The rostral anterior cingulate cortex (rACC) is involved in pain aversion (Cao et al., 2009). Researchers have reported that rACC damage can alleviate pain aversion but does not affect mechanical hypersensitivity (Shi et al., 2020). The rACC is involved in the rapid antidepressant effects of ketamine, and its gray matter volume is positively correlated with these effects (Herrera-Melendez et al., 2021). Neurons located in the V-VI layer of the rACC are the main projection neurons (Wu et al., 2009), which primarily consist of pyramidal neurons. Glutamatergic transmission plays a crucial role in processing the affective aspects of pain (Xiao et al., 2013). Therefore, we subsequently focused on the role of glutamatergic neurons in the rACC in the descending pain modulatory system and their relationship with pain and pain-related negative emotions.

Pain transmission is mainly regulated by the ascending and descending pain modulatory systems (Chen and Heinricher, 2019; Zhu et al., 2019; Kang et al., 2020). The midbrain ventrolateral periaqueductal gray (vlPAG), the major component of the descending pain modulatory system, plays a key role in inhibiting the upward nociceptive transmission of the spinal dorsal horn (Lau and Vaughan, 2014; Tobaldini et al., 2019). The activation of glutamatergic neurons in the vlPAG by optogenetic and chemogenetic methods can produce an analgesic effect (Samineni et al., 2017). Therefore, the vlPAG is highly correlated with pain. It is an important midbrain region involved in the suppression and promotion of endogenous pain (Cheriyan and Sheets, 2018) which bidirectionally modulates fear and anxiety, in addition to pain (Tovote et al., 2016; Frontera et al., 2020). Moreover, the vlPAG is a key midbrain structure related to defensive behavior that mediates anxiety and fear responses (Vázquez-León et al., 2018). Thus, both the rACC and vlPAG are highly correlated with pain-related negative emotions. However, it is not clear whether the circuit between the rACC and vlPAG can contribute to the modulation of hyperalgesia and pain-related negative emotions in spared nerve injury (SNI) mice models.

Clinically, in addition to analgesic drugs, antidepressant and anti-anxietic agents are used to treat chronic pain (Gilron et al., 2015). The combined use of these drugs can reduce pain sensation and relieve pain emotion. However, the efficacy of the combination of these drugs is limited, and the recurrence rate is high. The combined use of these drugs is also often associated with significant adverse drug reactions (Rocchio and Ward, 2021) such as drug addiction (Volkow and McLellan, 2016; Gao et al., 2021), adverse gastrointestinal reactions, and hypertension (Le et al., 2021). Electroacupuncture (EA) is widely used as an effective analgesic therapy in clinical practice. Both clinical and experimental studies have shown that EA can effectively relieve pain (Li et al., 2014; Lu et al., 2017), without drug addiction or adverse gastrointestinal reactions. Our previous studies have shown that EA can inhibit chronic inflammatory pain and pain-related anxiety-like behaviors in rats by increasing the expression of the neuropeptide S/neuropeptide S receptor system in the rACC (Du et al., 2020). Other researchers (Zhu et al., 2019) have shown that it can inhibit gamma-aminobutyric acid (GABA) neurons in the vlPAG through cannabinoid receptor 1 of the vlPAG and activate glutamatergic neurons to produce an analgesic effect. However, whether EA plays an analgesic role through the rACC*^Glu^*-vlPAG pathway is unclear.

This study aimed to explore whether EA can alleviate SNI-induced neuropathic pain and pain-related anxiety-like behaviors through the rACC*^Glu^*-vlPAG circuit. Herein, we used viral tracing and chemogenetic methods, combined with mechanical paw withdrawal thresholds (PWTs) assessment, the elevated plus maze (EPM) test, and the open field test (OFT), to detect the role of the rACC*^Glu^*-vlPAG circuit in these symptoms in SNI mice models.

## Materials and Methods

### Animals

All experiments were conducted using C57BL/6J adult male mice aged 8–10 weeks. The animals were obtained from the Laboratory Animal Center of Zhejiang Chinese Medicine University, accredited by the Association for Assessment and Accreditation of Laboratory Animal Care. The male mice were group-housed with five mice per cage that was covered with corn cob padding. Before the experiment, the mice were fed an adaptive diet for 1 week in a stable environment (temperature: 23–25°C, humidity: 40–60%). They were maintained under a 12-h light-dark cycle (lights on from 8:00 to 20:00), with food and water available ad libitum. Ventilation and air filtration systems were used throughout the study period. The experiment was conducted in accordance with the Guiding Opinions on the Ethical Treatment of Laboratory Animals issued by the Ministry of Science and Technology, PRC in 2006.

### Animal Models of Neuropathic Pain

All SNI mice models were anesthetized using isoflurane during the surgery. The left hind limb was shaved to expose the skin and sterilized with iodine and alcohol swabs. An incision was created above the midpoint of the line between the greater trochanter of the femur and the head of the tibia. The underlying tissue and muscle were opened via blunt dissection to expose the sciatic nerve trunk and branches. The peroneal and sural branches were tightly ligated with non-absorbent 6-0 sutures and transected below the ligature. Subsequently, a 2-mm section distal to the ligature was removed, while the tibial nerve was left intact. The incision was sutured layer-by-layer and disinfected with iodophor. Sham mice underwent the same surgery but without nerve ligation or severing.

### Virus and Trace Injection

The mice requiring stereotaxic brain injection received intraperitoneal anesthesia with 0.3% pentobarbital sodium and were fixed on a stereotaxic frame (RWD, 68025, Shenzhen, China). The temperature of the mice was maintained at 36°C using a heating pad (RWD, 69000, Shenzhen, China) and monitored using a thermostat during the surgery. A volume of 80 nL of virus was injected through glass microelectrodes connected to an infusion pump (WPI, UMC4, Sarasota, FL, United States) at a rate of 60 nL⋅min^–1^. The pipette remained at the injection site for 8 min at the end of the infusion to avoid virus overflow. The coordinates included the following three elements: dorsal-ventral (DV) from the brain surface, medial-lateral (ML) from the midline, and anterior-posterior (AP) from the bregma. According to Paxinos and Franklin’s The Mouse Brain in Stereotaxic Coordinates (Fifth Edition), the accurate injection location for the rACC (AP, +1.55 mm; ML, ±0.35 mm; DV, –0.85 mm) and the vlPAG (AP, –4.80 mm; ML, ±0.5 mm; DV, –2.15 mm) were determined.

For anterograde tracer virus injection in the rACC, a total of 10 mice received an anterograde tracer virus (AAV2/9-CAG-EGFP, 3.64 × 10^12^ vg/mL; BrainVTA, China) microinjection in the right rACC. For retrograde monosynaptic tracing, viruses (AAV2/R-CaMKIIα-EGFP, 5.24 × 10^12^ vg/mL; BrainVTA) were injected into the right vlPAG. After 3 weeks, the mice were anesthetized with an intraperitoneal injection of pentobarbital sodium and transcardially perfused with saline and 4% paraformaldehyde. Brain slices were prepared to record the GFP signals.

To investigate whether the rACC*^Glu^*-vlPAG pathway is involved in the regulation of hyperalgesia and anxiety-like behavior, we used chemogenetic techniques to specifically activate or inhibit this pathway. To specifically manipulate the glutamatergic neurons in the rACC output to the vlPAG, we injected AAV2/9-CaMKIIα-DIO-hM3D-mCherry at 3.04 × 10^12^ vg/mg (BrainVTA) and AAV2/9-CaMKIIα-DIO-mCherry at 5.35 × 10^12^ vg/ml (BrainVTA) into the right rACC and AAV2/R-CaMKIIα-Cre at 6.65 × 10^12^ vg/mL (BrainVTA) into the right vlPAG. We also injected AAV2/9-CaMKIIα-DIO-hM4D-mCherry at 3.38 × 10^12^ vg/mL (BrainVTA) and AAV2/9-CaMKIIα-DIO-mCherry at 5.35 × 10^12^ vg/mL (BrainVTA) into the bilateral rACC and AAV2/R-CaMKIIα-Cre at 6.65 × 10^12^ vg/mL (BrainVTA) into the bilateral vlPAG to inhibit the pathway. Clozapine-*N*-oxide (CNO) (1 mg/mL) (BrainVTA) was injected (2 mg/kg, intraperitoneally) into the mice at 7, 9, 11, 13, and 15 days after SNI. After the behavioral test on day 16, all mice were deeply anesthetized and perfused with 0.9% saline followed by 4% (w/v) paraformaldehyde. The brains were subsequently removed and post-fixed in 4% paraformaldehyde overnight at 4°C, dehydrated in sucrose (15 and 30%), and preserved at –80°C. Images of virus expression were obtained using a virtual slide microscope (VS120-S6-W; Olympus, Japan).

### Assessment of Anxiety-Like Behaviors

All behavioral tests were conducted in a dimly lit (∼20 lx) room. The mice were introduced to the behavioral test room where they were habituated for at least 1 day before testing. The experimental environment conditions were as follows: temperature: 23–25°C, humidity: 45–55%, and noise: < 40 dB. After injection with CNO or 0.9% saline for 30 min, the behavioral test was started. All behaviors were videotaped using a video tracking system.

#### Elevated Plus Maze Test

The EPM test was conducted at 15 days after SNI to evaluate anxiety-like behaviors among the mice. This test consisted of two closed arms (30 × 6 × 15 cm^3^), two opposing open arms (30 × 6 cm^2^), and a central platform (6 × 6 cm^2^). The open and closed arms were placed vertically in a cross. The maze was placed 35 cm above the floor. At the beginning of the experiment, the mice were placed on an open arm with their heads facing the intersection. After 30 s of adaptation, the activity of the mice was recorded for 5 min. After each test, the apparatus was washed with 75% alcohol to prevent olfactory cue bias.

#### Open Field Test

The OFT was conducted at 16 days after SNI. The open field was a 40 × 40 × 40-cm^3^ cuboid, uncovered wooden case, and each bottom was evenly divided into four equal parts, with a total of 16 small boxes. The outer 12 boxes were defined as the periphery region, and the middle four boxes were defined as the central region. In the experiment, the mice were placed at the center of the open field. After 30 s of adaptation, the activity of the mice was recorded for 5 min. After each test, the open field was washed with 75% alcohol to prevent olfactory cue bias.

### Von Frey Filament Test

The mechanical withdrawal threshold was measured using a von Frey filament in the middle of the left hind paw. The mice were placed individually in a single plexiglass chamber on a wire-mesh platform for detection. After an adaptive time of approximately 1 h, the filament probe was inserted, and the pressure was gradually increased. When the mice suddenly retracted, flinched, or licked their claws, the mechanical withdrawal response was positive. Three positive responses in five stimuli were defined as PWTs, and the interval of each measurement was more than 1 min. The PWTs were measured at baseline and 7 and 14 days after SNI.

### Electroacupuncture

Electroacupuncture was performed at 7, 9, 11, 13, and 15 days after SNI. We selected the “Zusanli” (ST36) and “Sanyinjiao” (SP6) acupoints of the bilateral hind limbs for EA. The mice were loosely immobilized and inserted with 0.16 × 7 mm acupuncture needles directly 5 mm into the bilateral Zusanli and Sanyinjiao acupoints. The needles were connected to a HANS acupoint nerve stimulator (HANS-200A Huawei Co., Ltd., Beijing, China). The stimulation parameters were as follows: frequency: 2 Hz, intensity: 0.1 mA, and time: 30 min. Except for the EA group, the sham and SNI groups were loosely immobilized. For sham EA treatment, mice were inserted with needles into the same acupoints (5 mm depth into ST36 and SP6) but without electrical stimulation.

### Immunohistochemistry

The mice were anesthetized with an intraperitoneal injection of sodium pentobarbital. They were then perfused with 0.9% saline, followed by 4% paraformaldehyde. The brains were extracted and placed in 4% paraformaldehyde at 4°C overnight and dehydrated using 15% (w/v) and 30% (w/v) sucrose until they sank. Coronal sections (30 μm) were cut using a cryostat frozen microtome (Thermo Fisher Scientific, NX50, United States). For immunofluorescence, the sections were rewarmed at 37°C for 1 h. They were placed on a shaker and washed with TBST six times for 10 min each time. The brain sections were incubated with 10% donkey blocking buffer at 37°C for 1 h. The sections were then incubated with primary antibodies, including rabbit anti-c-Fos (1:1000, ab190289, Abcam, United States), mouse anti-VGLUT1 (1:200, MAB5502, Millipore, United States), and mouse anti-VGLUT2 antibodies (1:200, MAB5504, Millipore, United States) at 4°C overnight. Subsequently, the sections were rewarmed for 1 h at 37°C, washed with TBST six times, and finally incubated with Alexa Fluor 488-conjugated secondary antibodies (715-545-151 and 711-545-152, Jackson Immunoresearch, West Grove, PA, United States) for 1 h at 37°C. The sections were then washed six times with TSBT and incubated with 4,6-diamidino-2-phenylindole (DAPI, ab104139, Abcam, United States) at the last stage. Thereafter, they were imaged and scanned using a digital pathological section scanner.

### Statistical Analysis

All experimental data were expressed as means ± standard errors of the means (SEMs). Two-way repeated-measures analysis of variance (rmANOVA), followed by the Tukey *post hoc* test, was used to statistically analyze the results of the PWTs assessment. One-way analysis of variance (ANOVA), followed by the LSD *post hoc* test was used to statistically analyze the results of the EPM test and OFT. Independent-sample *t*-tests were performed to evaluate c-Fos expression. Statistical significance was set at *P*-values of < 0.05.

## Results

### Anatomical Connection Between the Rostral Anterior Cingulate Cortex Glutamatergic Neurons and Ventrolateral Periaqueductal Gray

**FIGURE 1:**
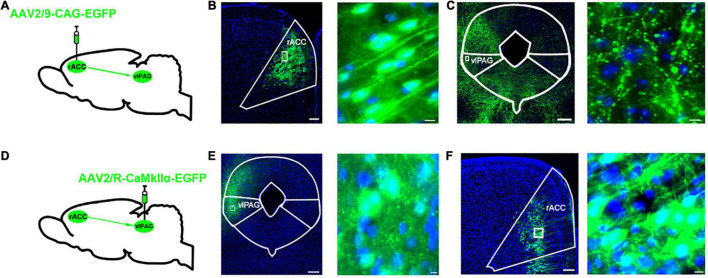
Dissection of the rACC*^Glu–^*vlPAG circuit. **(A)** Schematic diagram of AAV2/9-CAG-EGFP injection in the right rACC. **(B)** Representative images of right vlPAG-projecting neurons in the right rACC after 3 weeks (left) and local magnification of the injection site (right). Scale bar, 200 μm (left), 20 μm (right). **(C)** Representative images of right vlPAG fibers projecting from the right rACC. Scale bar, 200 μm (left), 10 μm (right). **(D)** Schematic diagram of AAV2/R-CaMKIIα-EGFP injection in the right vlPAG. **(E)** Representative images of the retrograde tracer virus injection site in the right vlPAG after 3 weeks (left). Scale bar, 200 μm (left), 10 μm (right). **(F)** Representative images of the glutamatergic neurons in the right rACC retrograde from the right vlPAG. Scale bar, 200 μm (left), 10 μm (right). rACC, rostral anterior cingulate cortex; vlPAG, ventrolateral periaqueductal gray.

To identify the anatomical connection between the rACC and vlPAG, we infused anterograde tracing viruses (AAV2/9-CAG-EGFP) labeled with neurons into the right rACC of C57 mice (Figure 1A). After 3 weeks, the EGFP-labeled neurons were observed in the right rACC (Figure 1B), while the EGFP-labeled fibers were observed in the right vlPAG (Figure 1C). Furthermore, a retrograde tracer virus (AAV2/R-CaMKIIα-EGFP) was injected into the right vlPAG (Figure 1D). After 3 weeks, the EGFP-labeled fibers were observed in the right vlPAG (Figure 1E), while the EGFP-labeled glutamatergic neurons were observed in the right rACC (Figure 1F). The results indicate a structural connection between the rACC*^Glu^* and vlPAG.

### Activation of the rACC*^Glu^*-vlPAG Pathway Leads to Hyperalgesia and Anxiety-Like Behaviors in Sham Mice

**FIGURE 2:**
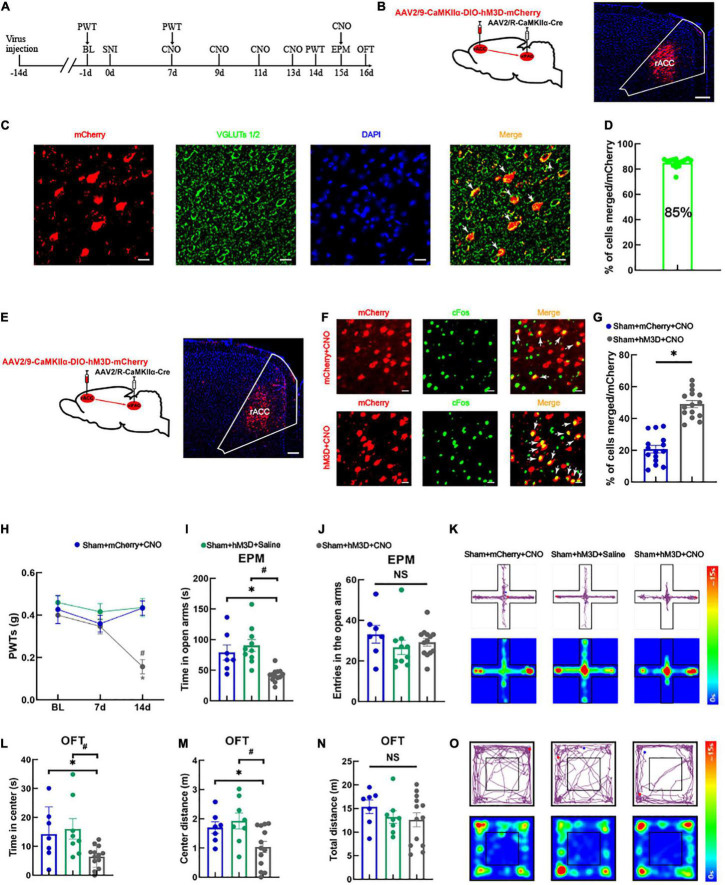
Specific activation of the rACC*^Glu^*-vlPAG neural circuit leads to hyperalgesia and anxiety-like behaviors in sham mice. **(A)** Schematic diagram of the experimental process. **(B)** Schematic diagram of chemical genetic virus injection (left) and representative image of the mCherry-labeled cells in the rACC after virus injection (right). Scale bar, 200 μm. **(C)** The mCherry (red) and glutamatergic neuron marker VGLUTs 1/2 (green) were co-localized in the rACC. Scale bar, 20 μm. **(D)** Percentage of mCherry and VGLUTs 1/2 co-localization (*n* = 15 slices from three mice). **(E)** Schematic diagram of chemogenetics (left) and representative image of viral expression within the right rACC (right). Scale bar, 100 μm. **(F)** Representative images of the glutamatergic neurons (red) in the rACC that co-localized with c-Fos. Scale bar, 20 μm. **(G)** Percentage of co-localization of glutamate neurons and c-Fos in each group (*n* = 15 slices from three mice, *P < 0.05). **(H)** Time course of the changes in the PWTs. **(I)** Time spent in the open arms in the EPM test. **(J)** There is no significant difference between the entries in the open arms covered by the mice among the three groups. **(K)** Representative movement trajectory diagram and activity heatmap of the sham + mCherry + CNO, sham + hM3D + saline, and sham + hM3D + CNO groups in the EPM test. **(L)** Time spent in the center area in the OFT. **(M)** Center distance covered by the mice in the OFT. **(N)** There is no significant difference between the total distance covered by the mice in the OFT among the three groups. **(O)** Representative movement trajectory diagrams and activity heatmaps in the sham + mCherry + CNO, sham + hM3D + saline, and sham + hM3D + CNO groups in the OFT. Data are presented as means ± standard errors of the means. *P < 0.05 vs. the sham + mCherry + CNO group; ^#^*P* < 0.05 vs. the sham + hM3D + saline group; NS, not significant. *n* = 7–13 mice per group in (H–N). PWT, paw withdrawal threshold; EPM, elevated plus maze; OFT, open field test; CNO, clozapine-*N*-oxide; SNI, spared nerve injury.

For the following experiment (schematic diagram of the experimental process was shown in Figure 2A), we manipulated the right vlPAG projecting glutamatergic neurons in the right rACC using a chemogenetic virus labeled with mCherry (Figure 2B). Approximately 85% of the vlPAG-projecting neurons labeled with mCherry in the rACC were immunoreactive for VGLUTs 1/2 (Figures 2C,D), indicating that these cells were glutamatergic neurons. We injected a Cre-dependent chemogenetic excitatory hM3D virus (AAV2/9-CaMKIIα-DIO-hM3D-mCherry) or a Cre-dependent control mCherry virus (AAV2/9-CaMKIIα-DIO-mCherry) into the right rACC and AAV2/R-CaMKIIα-CRE virus into the right vlPAG. Virus expression was detected in the right rACC after 3 weeks (Figure 2E). The excitatory role of the hM3D virus can be determined by comparing the co-localization percentage of the virus-labeled neurons with c-Fos between the control mCherry and hM3D (Figure 2F). Our analysis showed that the hM3D virus significantly activated the vlPAG-projecting glutamatergic neurons in the right rACC (Figure 2G). At 14 days after SNI, the PWTs of the sham + hM3D + CNO group significantly decreased compared with those of the sham + mCherry + CNO and sham + hM3D + saline groups [two-way rmANOVA: group: *F*_(2,66)_ = 3.941, *P* < 0.05; time: *F*_(2,66)_ = 11.98, *P* < 0.05; group × time: *F*_(4,90)_ = 4.485, *P* < 0.05, followed by Tukey *post hoc* test: *P* < 0.05, *P* < 0.05] (Figure 2H). The time spent in the open arms in the sham + hM3D + CNO group significantly decreased compared with that in the sham + mCherry + CNO and sham + hM3D + saline groups [one-way ANOVA: *F*_(2,27)_ = 12.309, *P* < 0.05, followed by the LSD *post hoc* test: *P* < 0.05, *P* < 0.05] (Figure 2I). The results of the entries in the open arms were not significantly different among the three groups [one-way ANOVA: *F*_(2,27)_ = 0.931, *P* > 0.05] (Figure 2J). Representative movement trajectory diagrams and activity heatmaps of the mice in the EPM test for each group are shown in Figure 2K. In the OFT, the spent time and travel distance in the central area in the sham + hM3D + CNO group significantly decreased compared with those in the sham + mCherry + CNO and sham + hM3D + saline groups [spent time: one-way ANOVA: *F*_(2,25)_ = 4.886, *P* < 0.05, followed by the LSD *post hoc* test: *P* < 0.05, *P* < 0.05, Figure 2L; travel distance: one-way ANOVA: *F*_(2,25)_ = 4.890, *P* < 0.05, followed by the LSD *post hoc* test: *P* < 0.05, *P* < 0.05, Figure 2M]. Meanwhile, the total distance traveled was not significantly different among the three groups [one-way ANOVA: *F*_(2,25)_ = 0.850, *P* > 0.05] (Figure 2N). Representative movement trajectory diagrams and activity heatmaps of the mice in the OFT for each group are shown in Figure 2O. These results show that specific activation of the rACC*^Glu^*-vlPAG circuit can lead to hyperalgesia and anxiety-like behaviors in sham mice.

### Inhibition of the rACC*^Glu^*-vlPAG Circuit Reduces Algesia and Anxiety-Like Behaviors in Spared Nerve Injury Mice Models

**FIGURE 3:**
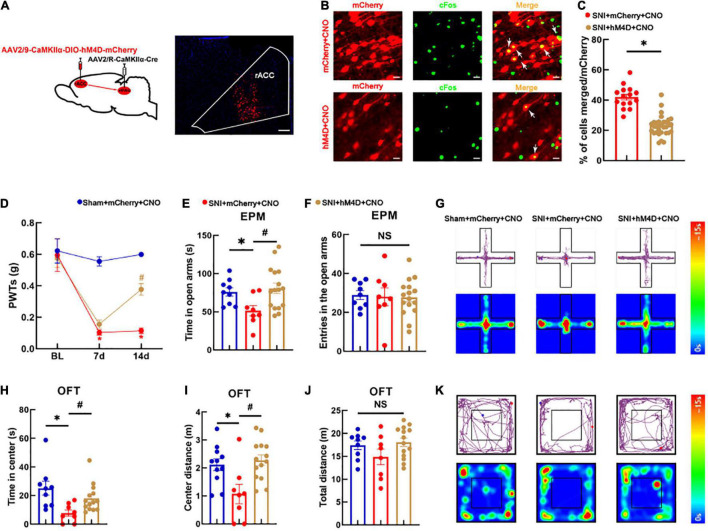
Specific inhibition of the rACC-vlPAG neural circuit relieves hyperalgesia and anxiety-like behaviors in SNI mice models. **(A)** The left side shows a schematic diagram of chemogenetics, and the right side shows a representative image of viral expression within the rACC. Scale bar, 100 μm. **(B)** Representative images of the glutamatergic neurons (red) in the rACC that co-localize with c-Fos. Scale bar, 20 μm. **(C)** Percentage of co-localization of the glutamatergic neurons and c-Fos in each group (*n* = 15 slices from three mice, *P < 0.05). **(D)** Time course of the SNI-induced changes in the PWTs. **(E)** Time spent in the open arms in the EPM test. **(F)** The entries in the open arms show no significant difference among the three groups. **(G)** Representative movement trajectory diagram and activity heatmap of the mice in the EPM test for each group. **(H)** Time spent in the center of the field by the mice in the OFT. **(I)** Center distance in the OFT. **(J)** There is no significant difference in the total distance covered by the mice in the OFT among the three groups. **(K)** Representative movement trajectory diagram and activity heatmap of the significant mice in the OFT for each group. Data are presented as means ± standard errors of the means. *P < 0.05 vs. the sham + mCherry + CNO group; ^#^*P* < 0.05 vs. the SNI + mCherry + CNO group; NS, not significant. *n* = 8–16 mice per group in **(D–F)**; *n* = 8–14 mice per group in **(H–J)**.

We next examined whether specific inhibition of the rACC*^Glu^*-vlPAG pathway could alleviate hyperalgesia and pain-induced anxiety-like behaviors in SNI mice models. The AAV-CaMKIIα-CRE virus was injected into the vlPAG bilaterally and the CaMKIIα-DIO-hM4D-mCherry virus into the rACC bilaterally. After 3 weeks, viral expression was detected in the rACC region of the brain (Figure 3A). We further examined the co-localization of the glutamatergic neurons and c-Fos (Figure 3B). Our analysis showed that the hM4D virus significantly inhibited the activity of the vlPAG-projecting glutamatergic neurons in the rACC (Figure 3C). As shown in Figure 3D, the SNI mice models displayed a significant reduction in their pain thresholds [two-way rmANOVA: group: *F*_(2,90)_ = 31.58, *P* < 0.05; time: *F*_(2,90)_ = 26.78, *P* < 0.05; group × time: *F*_(4,90)_ = 6.715, *P* < 0.05]. The *post hoc* analysis revealed no significant difference in the PWTs among the three groups before SNI (Tukey test, *P* > 0.05) (Figure 3D). On day 7 after SNI, the PWTs in the SNI + mCherry + CNO group significantly decreased compared with those in the sham + mCherry + CNO group (Tukey test, *P* < 0.05) (Figure 3D). After 14 days of modeling, the PWTs in the sham + mCherry + CNO group remained lower than those in the SNI + mCherry + CNO group (Tukey test, *P* < 0.05) (Figure 3D). Meanwhile, the PWTs in the SNI + hM4D + CNO group significantly increased compared with those in the SNI + mCherry + CNO group (Tukey test, *P* < 0.05) (Figure 3D). All groups were subjected to the EPM test. The time spent in the open arms among the SNI + mCherry + CNO group decreased compared with that among the sham + mCherry + CNO group [one-way ANOVA: *F*_(2,30)_ = 4.316, *P* < 0.05, followed by the LSD *post hoc* test: *P* < 0.05] (Figure 3E). The time spent in the open arms among the SNI + hM4D + CNO group increased compared with that among the SNI + mCherry + CNO group (LSD *post hoc* test: *P* < 0.05) (Figure 3E). The results of the entries in the open arms showed no significant differences among the three groups [one-way ANOVA: *F*_(2,30)_ = 0.044, *P* > 0.05, Figure 3F]. A representative movement trajectory diagram and an activity heatmap of the mice in the EPM test for each group are shown in Figure 3G.

The groups were also subjected to the OFT. The time spent in the center and center distance in the SNI + mCherry + CNO group decreased compared with those in the sham + mCherry + CNO group [one-way ANOVA: *F*_(2,30)_ = 3.695, *P* < 0.05, followed by the LSD *post hoc* test: *P* < 0.05, Figure 3H; one-way ANOVA: *F*_(2,30)_ = 6.537, *P* < 0.05, followed by the LSD *post hoc* test: *P* < 0.05, Figure 3I]. The time spent in the center and center distance in the SNI + hM4D + CNO group increased compared with those in the SNI + mCherry + CNO group [one-way ANOVA: *F*_(2,30)_ = 3.695, *P* < 0.05, followed by the LSD *post hoc* test: *P* < 0.05, Figure 3H; one-way ANOVA: *F*_(2,30)_ = 6.537, *P* < 0.05, followed by the LSD *post hoc* test: *P* < 0.05, Figure 3I]. There was no significant difference in the total distance of movement among the three groups [one-way ANOVA: *F*_(2,30)_ = 1.987, *P* > 0.05] (Figure 3J). Representative movement trajectory diagrams and activity heatmaps of the mice in the OFT for each group are shown in Figure 3K. The results indicate that specific inhibition of the rACC*^Glu^*-vlPAG neural circuit relieves hyperalgesia and anxiety-like behaviors in SNI mice models.

### Electroacupuncture Effectively Reduces Hyperalgesia and Anxiety-Like Behaviors in Spared Nerve Injury Mice Models

**FIGURE 4:**
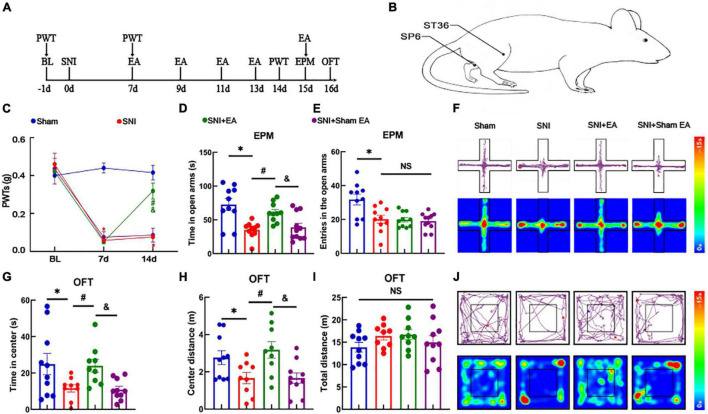
Electroacupuncture relieves hyperalgesia and anxiety-like behaviors in SNI mice models. **(A)** Schematic diagram of the experimental process. **(B)** Schematic diagram of the EA treatment. **(C)** Time course of the SNI-induced changes in the PWTs. **(D)** Time spent in the open arms by the mice in the EPM test. **(E)** The entries in the open arms show no significant difference among the three groups. **(F)** Representative movement trajectory diagram and activity heatmap of the mice in the EPM test for each group. **(G)** Time spent by the mice in the center of the field in the OFT. **(H)** Center distance in the OFT. **(I)** Total distance traveled in the OFT. **(J)** Representative movement trajectory diagram and activity heatmap of the mice in the OFT for each group. Data are presented as means ± standard errors of the means. *P < 0.05 vs. the sham group; ^#^*P* < 0.05 vs. the SNI group; ^&^*P* < 0.05 vs. the SNI + sham EA group; NS, not significant. *n* = 10–11 mice per group in **(C–E)**; *n* = 9–10 mice per group in **(G–I)**. EA, electroacupuncture.

We established a model of SNI-induced neuropathic pain and researched the effect of EA on model mice (schematic diagram of the experimental process and EA treatment were shown in Figures 4A,B). As shown in Figure 4C, the SNI mice model displayed a significant reduction in the pain thresholds [two-way rmANOVA: group: *F*_(3,111)_ = 61.84, *P* < 0.05; time: *F*_(2,111)_ = 155.5, *P* < 0.05; group × time: *F*_(6,111)_ = 31.54, *P* < 0.05]. The *post hoc* analysis revealed no significant difference in the PWTs among the four groups before SNI (Tukey test, *P* > 0.05) (Figure 4C). On day 7 after modeling, the PWTs in the SNI and SNI + EA groups significantly decreased compared with those in the sham group (Tukey test, *P* < 0.05, *P* < 0.05) (Figure 4C). At 14 days after modeling, the PWTs significantly differed between the SNI and sham groups (Tukey test, *P* < 0.05) (Figure 4C). The PWTs in the SNI + EA group significantly increased compared with those in the SNI group and SNI + sham EA group (Tukey test, *P* < 0.05, *P* < 0.05) (Figure 4C). The analysis showed that hyperalgesia induced by SNI lasted for at least 14 days. The SNI + EA group began to receive EA treatment (2 Hz, 0.1 mA) for 30 min once every 2 days on bilateral ST36 and SP6 acupoints located on the hind limbs of the mice. The SNI + sham EA group, with needles inserted into ST36 and SP6 acupoints but with no current stimulation, was used as a negative control.

The PWTs in the SNI + EA group significantly increased on day 14. These results indicate that 2 Hz EA can effectively alleviate hyperalgesia caused by SNI. In the EPM test, the time spent in the open arms among the SNI group decreased compared with that among the sham group [one-way ANOVA: *F*_(3,37)_ = 9.757, *P* < 0.05, followed by the LSD *post hoc* test: *P* < 0.05]. The time spent in the open arms among the SNI + EA group was longer than that among the SNI group and SNI + sham EA group [one-way ANOVA: *F*_(3,37)_ = 9.757, *P* < 0.05, followed by the LSD *post hoc* test: *P* < 0.05, *P* < 0.05, Figure 4D]. Meanwhile, the entries in the open arms in the sham group was longer than that among the SNI group [one-way ANOVA: *F*_(3,37)_ = 7.901, *P* < 0.05, followed by the LSD *post hoc* test: *P* < 0.05] (Figure 4E). A representative movement trajectory diagram and an activity heatmap of the mice in the EPM test for each group are shown in Figure 4F. In the OFT, the travel distance and time spent in the central area among the SNI group decreased compared with those among the sham group [one-way ANOVA: *F*_(3,34)_ = 4.088, *P* < 0.05, followed by the LSD *post hoc* test: *P* < 0.05, Figure 4G; one-way ANOVA: *F*_(3,34)_ = 4.694, *P* < 0.05, followed by the LSD *post hoc* test: *P* < 0.05, Figure 4H]. The time spent in the central area and center distance of the open field among the SNI + EA group were higher than those among the SNI group and SNI + sham EA group [one-way ANOVA: *F*_(3,34)_ = 4.088, *P* < 0.05, followed by the LSD *post hoc* test: *P* < 0.05, Figure 4G; one-way ANOVA: *F*_(3,34)_ = 4.694, *P* < 0.05, followed by the LSD *post hoc* test: *P* < 0.05, Figure 4H]. Meanwhile, the total distance traveled did not differ significantly among the four groups [one-way ANOVA: *F*_(3,34)_ = 1.224, *P* > 0.05] (Figure 4I). A representative movement trajectory diagram and an activity heatmap of the mice in the OFT for each group are shown in Figure 4J. The results indicate that depressive-like behaviors and hyperalgesia are reliably induced by SNI, and EA significantly relieves hyperalgesia and anxiety-like behaviors in SNI mice models.

### The Rostral Anterior Cingulate Cortex-Ventrolateral Periaqueductal Gray Circuit Underlies Electroacupuncture to Alleviate Neuropathic Pain but Not Anxiety-Like Behaviors

**FIGURE 5:**
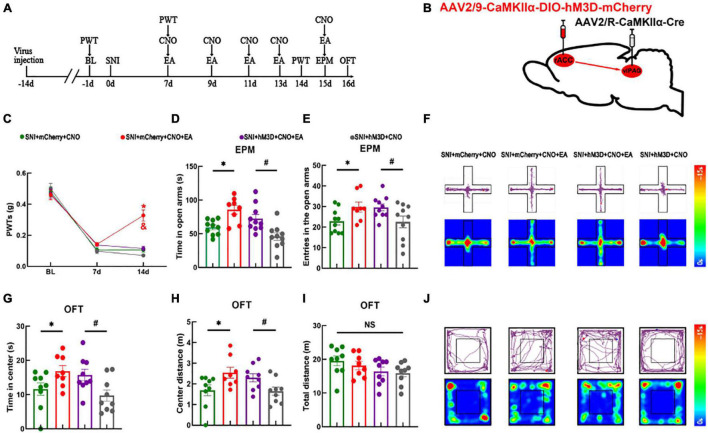
Electroacupuncture relieves pain sensation through the rACC*^Glu^*-vlPAG circuit, without ameliorating anxiety-like behaviors in SNI mice models. **(A)** Schematic diagram of the experimental process. **(B)** Schematic diagram of chemogenetics. **(C)** Time course of the SNI-induced changes in the PWTs. **(D)** Time spent in the open arms by the mice in the EPM test. **(E)** Entries in the open arms by the mice in the EPM test. **(F)** Representative movement trajectory diagram and activity heatmap of the SNI + mCherry + CNO, SNI + mCherry + CNO + EA, SNI + hM3D + CNO + EA, and SNI + hM3D + CNO groups in the EPM test. **(G)** Time spent in the center of the field by the mice in the OFT. **(H)** Center distance covered by the mice in the OFT. **(I)** Travel distance in the center area in the OFT. **(J)** Representative movement trajectory diagram and activity heatmap of the SNI + mCherry + CNO, SNI + mCherry + CNO + EA, SNI + hM3D + CNO + EA, and SNI + hM3D + CNO groups in the OFT. Data are presented as means ± standard errors of the means. *P < 0.05 vs. the SNI + mCherry + CNO group; ^#^*P* < 0.05 vs. the SNI + hM3D + CNO group; *P < 0.05 vs. the SNI + hM3D + CNO + EA group; NS, not significant. *n* = 10–12 mice per group in **(C–E)**; *n* = 9–10 mice per group in **(G–I)**.

The abovementioned experimental results showed that the rACC*^Glu^*-vlPAG pathway could regulate the mechanical withdrawal threshold and anxiety-like behaviors of mice in both physiological and pathological states. We demonstrated that activation of the rACC*^Glu^*-vlPAG pathway in the physiological state leads to pain and pain-related anxiety in mice. Moreover, we verified that EA could relieve hyperalgesia and anxiety-like behaviors in SNI mice models. Therefore, we specifically activated the rACC*^Glu^*-vlPAG pathway in SNI mice models to determine whether this activation would have an antagonistic effect on EA. We first injected the chemogenetic virus into the right rACC and right vlPAG brain regions (schematic diagram of the experimental process and chemogenetics were shown in Figures 5A,B). At 14 days after SNI, the PWTs of the SNI + mCherry + CNO + EA group were higher than those of the SNI + mCherry + CNO and SNI + hM3D + CNO + EA groups [two-way rmANOVA: group: *F*_(2,114)_ = 296.7, *P* < 0.05; time: *F*_(3,114)_ = 8.964, *P* < 0.05; group × time: *F*_(6,114)_ = 9.018, *P* < 0.05, followed by the Tukey *post hoc* test: *P* < 0.05, *P* < 0.05, Figure 5C]. The PWTs did not significantly differ between the SNI + hM3D + CNO group and SNI + hM3D + CNO + EA group (Tukey *post hoc* test: *P* > 0.05, Figure 5C).

The four groups were subjected to the EPM test. Compared with the SNI + mCherry + CNO group, the SNI + mCherry + CNO + EA group showed an increase in the time spent in the open arms and entries in the open arms [one-way ANOVA: *F*_(3,34)_ = 9.388, *P* < 0.05, followed by the LSD *post hoc* test: *P* < 0.05, Figure 5D; one-way ANOVA: *F*_(3,34)_ = 3.324, *P* < 0.05, followed by the LSD *post hoc* test: *P* < 0.05, Figure 5E]. Meanwhile, the SNI + hM3D + CNO group showed a decrease in the time spent in the open arms and entries in the open arms than the SNI + hM3D + CNO + EA group (LSD *post hoc* test: *P* < 0.05, Figure 5D; LSD *post hoc* test: *P* < 0.05, Figure 5E). A representative movement trajectory diagram and an activity heatmap of the mice in the EPM test for each group are shown in Figure 5F. The four groups that underwent the OFT showed similar results. Compared with the SNI + mCherry + CNO group, the SNI + mCherry + CNO + EA group showed an increase in the time spent in the center and center distance [one-way ANOVA: *F*_(3,30)_ = 3.710, *P* < 0.05, followed by the LSD *post hoc* test: *P* < 0.05, Figure 5G; one-way ANOVA: *F*_(3,30)_ = 3.689, *P* < 0.05, followed by the LSD *post hoc* test: *P* < 0.05, Figure 5H]. Compared with the SNI + hM3D + CNO group, the SNI + hM3D + CNO + EA group showed an increase in the time spent in the center and center distance (LSD *post hoc* test: *P* < 0.05, Figure 5G; LSD *post hoc* test: *P* < 0.05, Figure 5H). There was no significant difference in the total distance of the movement between the groups [one-way ANOVA: *F*_(3,30)_ = 1.302, *P* > 0.05] (Figure 5I). A representative movement trajectory diagram and an activity heatmap of the mice in the OFT for each group are shown in Figure 5J. The results indicate that specific activation of the rACC*^Glu^*-vlPAG pathway under pathological conditions antagonizes the analgesic effect of EA but not the anxiolytic effect of EA.

## Discussion

In this study, we found that specific activation of the rACC*^Glu^*-vlPAG pathway could lead to hyperalgesia and anxiety-like behaviors in sham mice in relation to changes in the synaptic plasticity of the glutamatergic neurons in the rACC output. Research has shown that changes in synaptic plasticity are closely related to the formation of pain, and synaptic plasticity is also the basis of central sensitization (Niu et al., 2020). Meanwhile, other researchers have pointed out that an increased expression of long-term potentiation in the rACC promotes pain-related anxiety through glutamate transmitter release (Bliss et al., 2016). Optogenetic activation of glutamatergic neurons in the rACC can cause pain-related anxiety-like behaviors (Sellmeijer et al., 2018; Elina et al., 2021).

The medial prefrontal cortex (mPFC) is a higher center of the brain and is involved in the regulation of pain, emotion, memory, and decision-making. In rodents, the mPFC can be divided into multiple regions, including the rACC, prelimbic cortex (PL), and infralimbic cortex (IL) (Porter and Sepulveda-Orengo, 2020). With the use of quinolinic acid to individually damage the rACC, PL, and IL, only bilateral damage to the PL alleviates hyperalgesia and anxiety-like behaviors in the complete Freund’s adjuvant (CFA) model (Wang et al., 2015), suggesting that the functions of these subregions are different. In addition, optogenetic activation of excitatory neurons in the contralateral PL reduces pain and anxiety-like behaviors induced by CFA, whereas inhibition is anxiogenic in naive mice (Wang et al., 2015). Another study showed that activation of the PL-PAG pathway increases the mechanical pain threshold, whereas inhibition decreases the mechanical pain threshold in naive mice. Moreover, activation of the PL-PAG pathway relieves hyperalgesia in tibial nerve transection mouse models (Drake et al., 2021). Another study (Yin et al., 2020) showed that the dorsal mPFC -ventrolateral periaqueductal gray (including the rACC-vlPAG and PL-vlPAG pathways) glutamatergic pathway positively regulates 5-HT neurons in the rostral ventromedial medulla, participating in a descending pain modulatory system from the cortex to the spinal cord. Specific activation of the dmPFC-vlPAG pathway by an optogenetic technique produced analgesic and anxiolytic effects in a common peroneal nerve ligation model. Our results showed that specific inhibition of the rACC*^Glu^*-vlPAG pathway alleviated hyperalgesia and anxiety-like behaviors in the SNI mice model. Previous literature and our results suggest that the vlPAG receives inputs from different brain regions and may regulate distinct circuits and downstream mechanisms, resulting in different functions. Furthermore, there are various types of neurons in the vlPAG, including glutamatergic, GABAergic, and 5-HT neurons (Chen et al., 2016). The activity of 5-HT neurons is tonically inhibited by local GABAergic neurons in the vlPAG (Millan, 2002; Chen et al., 2016). Based on our results, the rACC glutamatergic neurons may project to the vlPAG GABAergic neurons, and inhibition of the rACC*^Glu^*-vlPAG pathway may yield a feedforward inhibition from the GABAergic neurons to the 5-HT neurons in the vlPAG. This pathway is abnormally activated under pathological conditions, causing hyperalgesia and anxiety-like behaviors in SNI mice models. Under specific inhibition of the rACC*^Glu^*-vlPAG pathway, the descending facilitation effect was weakened, and the descending inhibition effect was enhanced, while hyperalgesia and anxiety-like behaviors also improved. We suggest that the PL modulates the vlPAG in the dmPFC-vlPAG pathway system and may play a key role in this pathway.

The therapeutic effect of EA on neuropathic pain has been supported by both clinical and experimental studies. A study showed that 2 Hz bilateral EA at the Zusanli and Sanyinjiao acupoints in SNI mice models significantly reduced SNI-induced chronic neuropathic pain (Xia et al., 2019). Further, EA can reduce the intensity of intractable pain and improve the emotional disturbances caused by pain (Kim et al., 2019). It is a valid treatment for pain and anxiety in clinical settings (Errington-Evans, 2012; Amorim et al., 2018). In prior results, we have found the inhibition of rACC*^Glu^*-vlPAG circuit or EA treatment could alleviate pain and related anxiety-like behaviors. To confirm whether EA exerts an analgesic effect and relieves anxiety through inhibiting the rACC*^Glu^*-vlPAG circuit, we activated the rACC*^Glu^*-vlPAG circuit in SNI mice models and then treated the mice with EA. Our results showed that EA could reduce neuropathic pain sensitization, which was reversed by activating the rACC*^Glu^*-vlPAG pathway. While anxiety-like behaviors were relieved by EA, the anxiolytic effect could not be reversed by activating the rACC*^Glu^*-vlPAG pathway. In contrast, our previous study has revealed that inhibition of the rACC*^Glu^*-thalamus circuit alleviated anxiety-like behaviors in CFA rat models but did not affect the mechanical pain threshold (Shen et al., 2020). Furthermore, activation of the rACC*^Glu^*-thalamus pathway specifically reversed the anxiolytic effect of EA in CFA rat models but had no effect on pain sensation. In summary, the analgesic effect of EA can be antagonized by the activated rACC*^Glu^*-vlPAG circuit, while the anxiolytic effect can be antagonized by the activated rACC*^Glu^*-thalamus circuit. This may be attributed to several factors, including the following: (1) During chronic pain, emotions and pain sensations are separated. EA may produce an analgesic effect through the rACC*^Glu^*-vlPAG pathway, rather than by intervening in negative emotions. Even if the rACC*^Glu^*-vlPAG circuit is activated, EA may exert an anti-negative emotional effect through other circuits; (2) The rACC*^Glu^*-vlPAG pathway is not the key circuit for EA to regulate pain-induced negative emotions in chronic neuropathic pain.

Electroacupuncture is an effective and safe alternative therapy for treating anxiety disorders (Errington-Evans, 2012). Studies have shown that EA at the Zusanli and Sanyinjiao acupoints can reduce the mechanical and thermal pain sensitivities of inflammatory pain induced by CFA in mice models (Wan et al., 2021). Acupuncture of the Zusanli acupoint can improve motor function and anxiety-like behavior in Parkinson’s disease mice models (Jang et al., 2020). Moxibustion at the Sanyinjiao acupoint can improve pain and emotional disorders in patients with primary dysmenorrhea (Liu et al., 2020). Our previous studies have shown that the combination of the Zusanli and Sanyinjiao acupoints is the dominant acupoint combination for the treatment of pain and depression comorbidity (Wu et al., 2015). Pain sensitization and anxiety-like behaviors were reduced in the SNI mice models with the application of EA (Zusanli and Sanyinjiao acupoints) at 2 Hz for 30 min in our study. When the rACC*^Glu^*-vlPAG circuit was activated, the analgesic effects of EA and not the anxiolytic effects were antagonized. This suggests that EA can modulate pain sensation by modulating the inhibition of the rACC*^Glu^*-vlPAG circuit, but not anxiety-like behaviors.

In conclusion, we demonstrated a novel circuit mechanism in which the rACC*^Glu^*-vlPAG circuit plays an important role in pain and anxiety induced by chronic neuropathic pain. Moreover, we found that EA induces analgesic but not anxiolytic effects via the rACC*^Glu^*-vlPAG pathway.

## Data Availability Statement

The raw data supporting the conclusions of this article will be made available by the authors, without undue reservation.

## Ethics Statement

The animal study was reviewed and approved by the Animal Ethics Committee of Zhejiang Chinese Medical University (ZSLL, 2017-183). The animals were obtained from the Laboratory Animal Center of Zhejiang Chinese Medicine University, accredited by the Association for Assessment and Accreditation of Laboratory Animal Care (AAALAC).

## Author Contributions

XZ, YLX, and ZS performed the data analysis and applied the chemogenetic method. HZ and SX performed the surgeries. YZ and MW designed the experiment protocols. YC performed the paw withdrawl threshold testing. BL and JL performed the manuscript writing. ZW performed the open field test. YYX and XH performed the immunostaining. JS performed the electroacupuncture treatment. JD performed the statistical analysis. All authors contributed to the article and approved the submitted version.

## Conflict of Interest

The authors declare that the research was conducted in the absence of any commercial or financial relationships that could be construed as a potential conflict of interest.

## Publisher’s Note

All claims expressed in this article are solely those of the authors and do not necessarily represent those of their affiliated organizations, or those of the publisher, the editors and the reviewers. Any product that may be evaluated in this article, or claim that may be made by its manufacturer, is not guaranteed or endorsed by the publisher.
